# From classical mendelian randomization to causal networks for systematic integration of multi-omics

**DOI:** 10.3389/fgene.2022.990486

**Published:** 2022-09-15

**Authors:** Azam Yazdani, Akram Yazdani, Raul Mendez-Giraldez, Ahmad Samiei, Michael R. Kosorok, Daniel J. Schaid

**Affiliations:** ^1^ Center of Perioperative Genetics and Genomics, Brigham Women’s Hospital, Harvard Medical School, Boston, MA, United States; ^2^ Health Science Center at Houston, McGovern Medical School, Division of Clinical and Translational Sciences, University of Texas, Houston, TX, United States; ^3^ Biostatistics and Computational Biology Branch, National Institute of Environmental Health Sciences, Durham, NC, United States; ^4^ Division of Pulmonary Medicine, Boston Children’s Hospital, Boston, MA, United States; ^5^ Department of Biostatistics, University of North Carolina at Chapel Hill, Chapel Hill, NC, United States; ^6^ Department of Quantitative Health Sciences, Mayo Clinic, Rochester, MN, United States

**Keywords:** systems biology, causal networks, stability of causal networks, principles of mendelian randomization, classical MR, systems approach, multiomic integration

## Abstract

The number of studies with information at multiple biological levels of granularity, such as genomics, proteomics, and metabolomics, is increasing each year, and a biomedical questaion is how to systematically integrate these data to discover new biological mechanisms that have the potential to elucidate the processes of health and disease. Causal frameworks, such as Mendelian randomization (MR), provide a foundation to begin integrating data for new biological discoveries. Despite the growing number of MR applications in a wide variety of biomedical studies, there are few approaches for the systematic analysis of omic data. The large number and diverse types of molecular components involved in complex diseases interact through complex networks, and classical MR approaches targeting individual components do not consider the underlying relationships. In contrast, causal network models established in the principles of MR offer significant improvements to the classical MR framework for understanding omic data. Integration of these mostly distinct branches of statistics is a recent development, and we here review the current progress. To set the stage for causal network models, we review some recent progress in the classical MR framework. We then explain how to transition from the classical MR framework to causal networks. We discuss the identification of causal networks and evaluate the underlying assumptions. We also introduce some tests for sensitivity analysis and stability assessment of causal networks. We then review practical details to perform real data analysis and identify causal networks and highlight some of the utility of causal networks. The utilities with validated novel findings reveal the full potential of causal networks as a systems approach that will become necessary to integrate large-scale omic data.

## Introduction

Due to recent technological advances, data acquisition of molecular components on large scales and in multiple omics capacities has been realized. Yet, advanced analytic methods are desperately needed to systematically integrate these data to facilitate discoveries and improved understanding of the biological process that impacts omics health and disease. Systematic analysis refers to the simultaneous analysis of all data in the study while considering their interconnectivity/dependency. Identifying the underlying relationships among molecular entities as a network provides insights into complex processes that would not be revealed by focusing on individual entities in isolation (Barabasi and Oltvai, 2004; Bebek et al., 2012). Such an analysis requires the incorporation of further relevant biological information (Ainsworth et al., 2017).

Causal networks, as a systematic analysis of data, are ideally suited for analyzing multi-omics and heterogeneous data sets to reveal the role of entities individually or as a module in a system (e.g., an omic, such as metabolomics). Using the principles of MR on a genome-wide scale and integrating genetics with other omic data allow researchers to relate information at different levels of omic data in a cohesive analytic framework and possibly uncover the underlying relationships that represent molecular networks (Badsha and Fu, 2019a; Ahangaran et al., 2019). Causal networks not only represent the connectivity among observations but also facilitate extracting causality from observational data (Holmes et al., 2017; Dorvash et al., 2020; Hackett et al., 2020; Khan et al., 2020).

Identifying causality through classical MR has received attention in biomedical research. The characteristic of this framework is hypothesis-driven with a focus on a small set of entities with known underlying relationships (known causal diagram) (Richmond et al., 2016). In modern biomedical research, i.e., large-scale omic data, however, there are several hundred or thousands of entities, and there is limited knowledge about interconnectivity among them. Causal networks are pragmatic to address the challenges of large-scale omics.

We here review the integration of classical MR and causal networks which seem mostly as two distinct branches of statistics. Here, we first briefly review some recent developments in the classical MR framework then, we discuss the identification of causal networks, evaluation of the underlying assumptions, and introduce some tests to assess the stability of the networks. We also review practical steps to identify causal networks on real data and review some utilities of causal networks, such as the identification of molecular regulatory sub-networks and the identification of molecules with an essential role in the system under study.

### An overview of mendelian randomization

To estimate causal relationships when experiments cannot be controlled or randomized, which is often the case for biomedical studies, statistical regression models are frequently used by regressing a response variable on an explanatory variable. However, regression models can give biased results when an explanatory variable is correlated with the regression model’s error term. To overcome this limitation, instrumental variables (IV) can be used, but with strong assumptions. A valid IV induces changes in the explanatory variable but not the response of interest other than through the explanatory variable, hence allowing identification of the causal effect of the explanatory variable on the response variable. The basis of MR is the use of IVs, as discussed extensively in the literature (Sanderson et al., 2022). In biomedical studies, genetic variants are frequently used as IVs because of assumptions of Mendelian genetics: random mating of parents and random transmission of alleles from parents to offspring. An IV is valid under the following assumptions (Bowden et al., 2015):

1. IV is associated with an explanatory variable conditional on other covariates in the model.
2. IV is not associated with unmeasured confounders.
3. IV is not associated with response conditional on the explanatory variable and unmeasured confounders.

In MR applications, when a genetic variant affects response via a different biological pathway from the explanatory variable, IV assumptions could be violated (i.e., a pleiotropic effect). Satisfying the second and third IV assumptions means a lack of pleiotropic action of IV , neither through the unmeasured confounder (i.e., correlated pleiotropic effect) nor directly (i.e., uncorrelated pleiotropic effect) (Xue et al., 2021). In the case of multiple independent genetic instruments for an explanatory variable, lack of pleiotropy can be replaced with the weaker Instrument Strength Independent of Direct Effect (InSIDE) assumption (Bowden et al., 2015): If there is no correlation between the genetic associations with the explanatory variable and the genetic associations with the response, the IV assumption is satisfied. To identify IV s with pleiotropic effects see the test heterogeneity in dependent instruments (Gao et al., 2016). Some recent efforts to relax MR assumptions and account for confounding due to pleiotropy are based on plurality validation of IV s: in large samples, while (Wald) ratio estimates of the target causal effect from invalid IV s will take different values, ratio estimates from all valid IV s should approach the true causal effect and thus, the valid IV s form the largest group of SNPs among all the groups giving different ratio estimates (Xue et al., 2021). For models based on plurality validation see e.g., constrained maximum likelihood and model averaging (Xue et al., 2021), MR mixture (Qi and Chatterjee, 2019), and MR-cause (Morrison et al., 2020). In addition to pleiotropy, another confounding factor in the summary-statistics MR approach is sample structure, such as population stratification and sample overlap, which needs to be considered, see (Hu et al., 2022).

### Some recent progress in the classical MR framework

One of the recent MR developments in the classical MR framework includes applications with several explanatory variables, called multivariable MR. Multivariable MR enables estimating the effects of multiple individual explanatory variables (primary and secondary explanatory variables) on one response to avoid violation of MR assumptions due to secondary explanatory variables being the confounders of the primary explanatory variable and response relationship (Porcu et al., 2019), Figure 1A. One approach to estimate the effects is the following. First, regress each explanatory variable (X) on the IV to estimate a predicted explanatory variable $\hat{X}$ . Then, regress the response on the predicted explanatory variables $\hat{X}s$ , the regression coefficients are called the causal effects of explanatory variables on response,

$$\hat{X_{h}}=\alpha_{\;h}\;{IV}_{h},\;h=1,\ldots ,n,$$ (1)

$$Response=\beta_{0}+\beta_{1}\;\hat{{\;X}_{1}}+\beta_{2}\;\hat{{\;X}_{2}}+\ldots +\beta_{n}\;\hat{{\;X}_{n}}+e.$$ (2)

**FIGURE 1:**
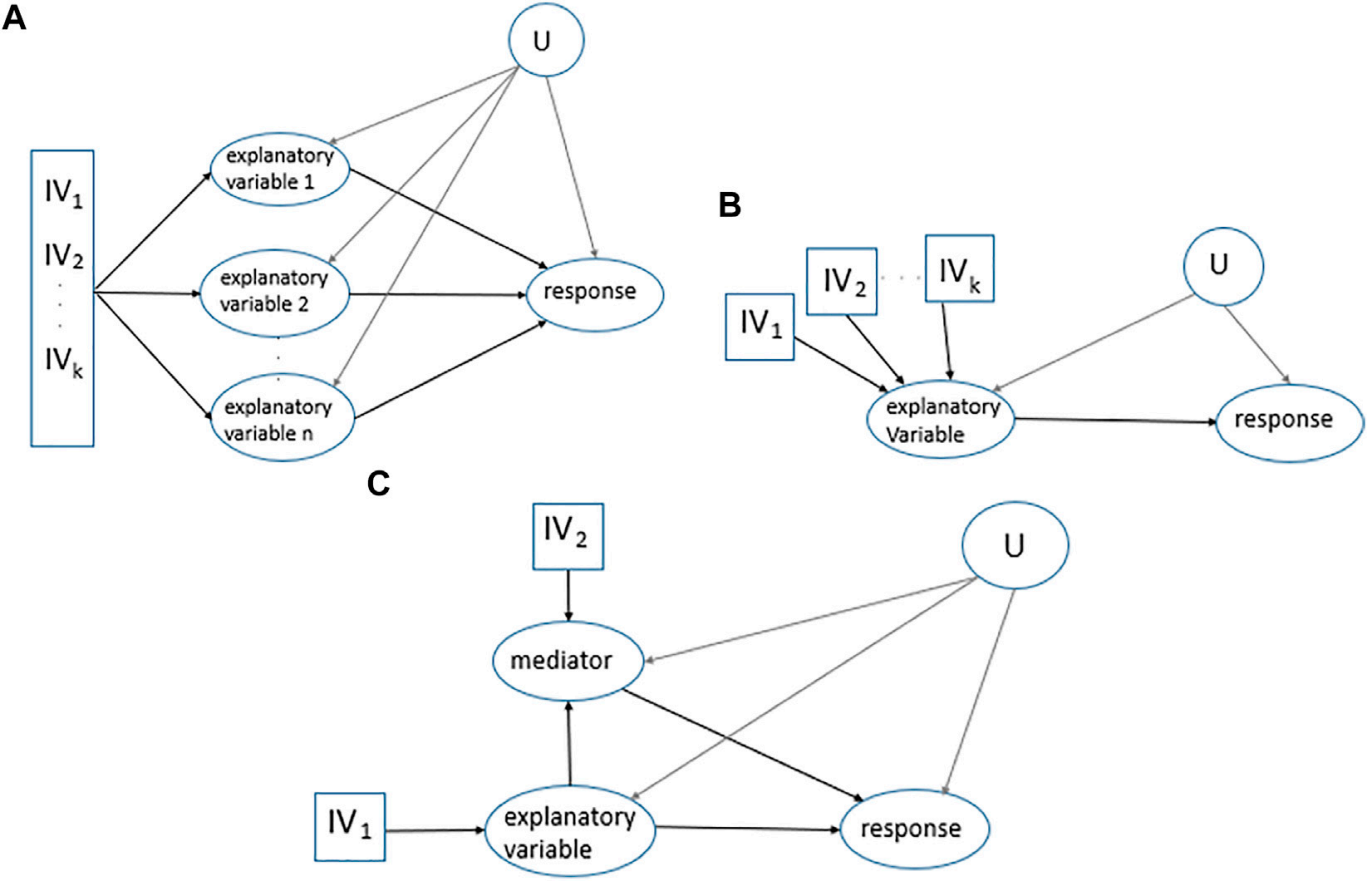
MR applications. **(A).** Multivariable MR. Multiple IV s for multiple explanatory variables of the same response to estimate the direct effect of each explanatory variable on the response. *U* stands for a set of confounders. **(B).** Multiple uncorrelated IV s. Multiple uncorrelated IV s for one explanatory variable to predict significant variation in the explanatory variable, satisfy a robust relationship between the IV and the explanatory variable. **(C).** Two-step MR for mediation analysis. In the case that there is a mediator, considering two IV s (one for the explanatory variable and one for the mediator) facilitates measuring the direct effect of the explanatory variable *X* on the response.

In Eqs 1 and 2 with the assumption of additive effects, $\hat{X}$ is estimating the value of the explanatory variable ( X ) using IV , α is the effect of IV on the explanatory variable X , coefficients β represent the causal effect of the explanatory variables X on the response, *n* is the number of explanatory variables in the multivariable MR.

The application of multiple uncorrelated IV s is suggested to increase the power of the IV approach to estimate the explanatory variable and as a result, to assess the relationship of the explanatory variable with the response, Figure 1B. This leads to predicting the explanatory variable as the following,

$$\hat{X}=\alpha_{1}\;{IV}_{\;1}+\ldots +\alpha_{k}\;{IV}_{k}.$$ (3)

Since the instruments are uncorrelated, the variation explained by each of the instruments is independent from the other.

In mediation analysis, the interest is in the contribution of variables that lie on the causal pathway from an explanatory variable to a response, Figure 1C. In this case, two-step MR is often applied which is a combination of two univariate MRs, estimating the causal effect of the explanatory variable on the mediator and then estimating the causal effect of the mediator on the response (Sanderson et al., 2019). Different causal effects including direct, indirect, and total effects in Figure 1C are as follows:

τ corresponds to the effect of explanatory variable X on the mediator: $mediator=\tau_{\;0}+\tau \;{\hat{X}}_{\;{IV}_{1}}+e^{\prime },$ (4)

γ corresponds to the effect of the mediator on response: $response=\gamma_{\;0}+\gamma \;{\hat{M}}_{\;{IV}_{2}}+e^{\prime \prime },$ (5)

φ Corresponds to the direct effect of explanatory variable X on response: φ=β−τ γ (6) where β stands for the total effect of the explanatory variable on response calculated as $Response=\beta_{\;0}+\beta \;{\hat{X}}_{\;{IV}_{1}}+e$ . Here ${\hat{X}}_{\;IV}$ is the predicted value of explanatory variable X by IV , i.e., variation in the explanatory variable explained by IV .

**FIGURE 2:**
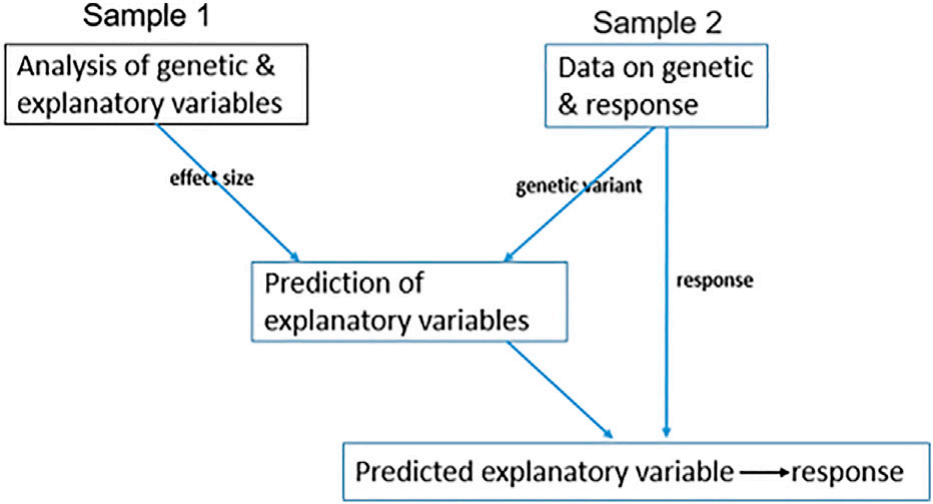
Two-sample MR. A diagram representing the application of two-sample MR when data on IV, explanatory variable, and response are not available for all samples. Sample 1 has genetic and explanatory variable records; therefore, we measure the effect size of genetic variants on the explanatory variable. Sample 2 has genetic variant and response records and not explanatory variable measurements, therefore, to estimate the genetic variation of any explanatory variable, we use the effect size from sample 1. Then, we estimate the causal relationship between the genetically estimated explanatory variable and response.

Due to high measurement costs or lack of appropriate biospecimens, data on IV , explanatory variable, and response might not be available for all participants. In this setting, to infer a causal relationship between an explanatory variable and a response, two-sample MR is applied, where one sample has data on the genetic and explanatory variable, and the other has data on genetic and response, e.g., (Gao et al., 2016). The algorithm for a two-sample MR application is provided in Figure 2. One way to predict the explanatory variable in sample two is

$$\hat{X}=\overset{k}{\underset{i=1}{\sum }}\beta_{{IV}_{i,\;sample1}}*{IV}_{i,sample2},$$ (4)

where $\hat{X}$ stands for prediction of the explanatory variable for sample two; *k* stands for the number of uncorrelated IVs, $\beta_{{IV}_{i,\;sample1}}$ stands for the effect size of the $i^{th}$ genetic variant with a significant association with the explanatory variable in sample 1, and ${IV}_{i,sample2}$ is the corresponding genetic variant in sample 2.

This approach can also be applied to summary statistics of both samples without having the individual levels (Lawlor, 2016; Zhu et al., 2016). In this setting, the causal effect of the explanatory variable on response is estimated by the effect of genetic variants on response in sample two divided by the effect of genetic variants on the explanatory variable in sample 1.

The application of summary statistics and two-sample studies are common for wide association studies, such as transcriptomic/phenotypic wide association studies, where the association of the predicted-explanatory variable and the response of interest is tested. Two-sample studies based on summary statistics are also used in colocalization, where we estimate the probability of the same signal for GWAS and the study of quantitative trait loci (QTL), such as expression or metabolite QTL. However, in these studies, the MR assumptions, especially the lack of pleiotropic effect, are not assessed. Otherwise, these studies will be the same as MR studies, see (Barfield et al., 2018).

### The transition from the classical MR framework

The applications above assume that a causal diagram is specified, i.e., the role of each component such as being a mediator, explanatory variable, or response is specified in *a priori* in a small set of variables, and the interest is finding an individual cause of a specific response. Even in multivariable MR when multiple explanatory variables are considered, the interest is in finding the individual causes of a response and not revealing the underlying relationships among the entities in the study. Therefore, the classical MR framework is hypothesis-driven (Richmond et al., 2016), which is a major limitation of the classical MR framework to address questions in modern biomedical studies where we have limited knowledge about relationships among entities, we do not know which entity is the response variable and which is the explanatory variable. In other words, in modern biomedical studies, the causal diagrams are unknown.

**FIGURE 3:**
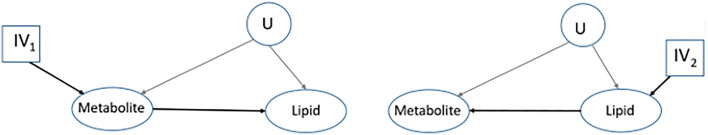
A transition from the classical MR framework. Interest is in finding the causal relationship between a metabolite and a lipid where we do not know which one is the response. Two of the possible causal diagrams are represented and each one will be assessed separately to select the most likely causal diagram.

To overcome this limitation of the classical MR framework, one of the early proposals was to consider all possible causal diagrams for the set of entities in the study, then, investigate each one independently using a statistical method, and finally, select the most likely causal diagram, Figure 3A, (Shin et al., 2014; Wittenbecher et al., 2022).

This approach is challenging computationally and statistically because when the number of entities in the study increases, the number of possible causal diagrams grows exponentially.

### Causal networks

Unlike classical MR framework, systems approaches such as causal networks deal with all entities under study at the same time. Causal networks are systematic analyses of data where connections among entities (nodes in the network) are essential to the conclusions. In this framework, each entity can be an explanatory variable, mediator, confounder, as well as a response at the same time. The key feature of causal networks is being discovery-based, and suitable for handling large-scale data, where we have limited knowledge about the underlying interconnectivity. There are different applications of systematic analysis of omics including causal networks (Zhu et al., 2012; Franzén et al., 2016; Broumand and Dadaneh, 2018; Ahangaran et al., 2019; Ahangaran et al., 2020; Yazdani et al., 2020; Gerring et al., 2021). For instance one of the early applications is the integration of genetic variants, metabolites, gene expressions, and proteins on yeast data to identify the underlying molecular networks (Zhu et al., 2012). Another example is the identification of different patterns of gene expression for patients with coronary artery disease (Franzén et al., 2016). More recently, the causal network successfully identified genes that are differentially regulated in schizophrenia-cases versus controls and found essential genes for human brain functions (Yazdani et al., 2020).

Causal networks can be based on Bayesian networks augmented with the principles of MR (Aten et al., 2008; Yazdani et al., 2016a; Badsha and Fu, 2019b; Howey et al., 2020). For details of the causal-network identification, see Supplementary, for a recent review of methods see (Ainsworth et al., 2017; Ghassami et al., 2017; Hu et al., 2018; Glymour et al., 2019), and for a comparison of MR performance and causal networks in both real and simulated data see (Howey et al., 2020). The MR approaches for causal network identification can be different for different data types (e.g., different omics). For entities whose levels are controlled by one or two local single nucleotide polymorphisms, we can use the related QTLs as IV s (Tsamardinos et al., 2006; Yazdani et al., 2016b). Otherwise, the use of a polygenic approach may facilitate the identification of IV s (Burgess et al., 2017; Yazdani et al., 2019; Yazdani et al., 2020). In the polygenic approach, we extract information from genetic variants to generate IV s (instead of using natural genetic variants) which can be carried out using principal component analysis or multiple correspondence analysis (Abdi and Valentin, 2007). Polygenic factors explain a large amount of genetic variation and thus have the potential to generate a stronger association with explanatory variables (Yazdani et al., 2016a). This approach prevents spurious estimates and increases the accuracy of findings compared to the cases where too many genetic variants are used. This approach also prevents highly sensitive estimates due to ignoring a majority of data and using a few genetic variants (Burgess et al., 2017). Extracting information from the genome and therefore generating many IV s provides an opportunity to allocate multiple independent IV s to each explanatory variable and increase the power of the MR analysis (Pierce et al., 2011) and identify causal networks on a large scale (Yazdani et al., 2016b).

### Causal networks and the evaluation of the IV assumptions

Causal networks are in the framework of Causality and the underlying assumptions to infer causality are the same as classical MR. The application of invalid IV s results in unstable causal networks. Therefore, for causal network identification, we not only embed the MR assumptions in the algorithms but also assess the stability of causal networks after identification and determine if the MR assumptions are violated in one or some parts of the networks.

In the constraint-based algorithms, causal networks are built upon conditional independence and simultaneous assessment of the lack of pleiotropic effect (the effect of IV on response is only through the explanatory variable). The causal relationship $M_{i\;}\to M_{j}$ is concluded if the property 7) is satisfied which explains that the effect of IV on response $M_{j}$ is only through the explanatory variable $M_{i}$ ,

$$M_{j}\;⊥\;IV\;|\;M_{i},\;$$

i.e., ${p(M\;}_{j}\;,\;IV\;|\;M_{i})=p\left(M_{j}|\;M_{i}\right)$ , (5)

And the deterministic representation of (7) is

$$M_{j}=f\left(M_{i},\;IV,U\right)\equiv {\;M}_{j}=f\left(M_{i},U\right),\;$$ (6)

where all factors that affect variable $M_{j}$ when variable $M_{i}$ is held constant is confined in *U*. In the property $M_{j}\;⊥\;IV\;|\;M_{i},$ the notation “ ${⊥}^{“}$ stands for statistical independence.

If this property is not satisfied, the variable used as IV does not qualify to investigate the causal relationship ${\;M}_{i}\to M_{j}$ , and as a result, will not be included in the analysis.

As reviewed above, the validity of IV s in causal networks is correspondence to causal network stability. In addition to the tests that we will introduce in the following, minimizing Hamming distance is one of the well-established assessments for the stability of the networks (Tsamardinos et al., 2006; Norouzi et al., 2012). Using Hamming distance, only robust connections remain in the network including IV - explanatory variable connections. Assessing the strength of IV connections using Hamming distance and pleiotropy assessment in Eq. 7 are implemented in the Genome granularity Directed acyclic Graph (G-DAG) algorithm to identify causal networks (Yazdani et al., 2016a).

We here introduce some tests to assess the stability of an identified causal network. Understanding the tests requires some background in basic concepts for causal network exploration, such as the “graphically” identification of cofounders using the “back-door” criterion, see Supplementary or (Pearl, 2011; Yazdani A, 2015). Due to some technical notations, the formal descriptions are provided in Supplementary.

**Confounding-equivalent Test**. Assume we are interested in the effect of X on Y , as two entities, in an identified causal network. Two sets, $S_{1}$ and $S_{2}$ are confounding-equivalent (relative to X and Y ), if the following equality holds for every x and y :

$$\underset{S_{1}=s_{1}}{\sum }P\left(Y=y\;|X=x,S_{1}=s_{1}\right)P\left(S_{1}=s_{1}\right)=\underset{S_{2}=s_{2}}{\sum }P\left(Y=y\;|\;X=x,S_{2}=s_{2}\right)P\left(S_{2}=s_{2}\right).$$

This equality guarantees that, adjusting for either set $S_{1}$ or $S_{2}$ would produce the same asymptotic bias relative to the target quantity, which is the identification of the effect X on Y .

**FIGURE 4:**
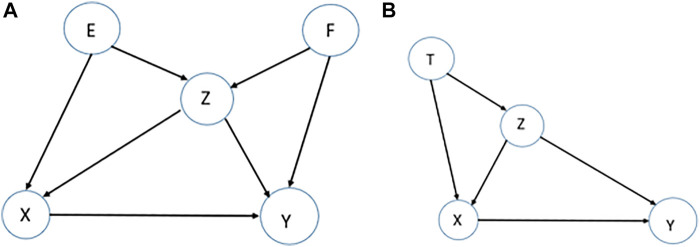
Examples for stability tests. **(A).** To assess the effect of {X} on {Y} in this causal network, there are two equivalent sets of confounders {E,Z } and {F, Z }, which means considering either of the sets, the study of the effect {X} on {Y} is unconfounded and the effect does not vary significantly (Confounding-equivalent Test). **(B).** To assess the effect of {X} on {Y} in this causal network, {Z} is the confounder. Therefore, knowing the value of variable T does not change estimating the effect of {X} on {Y} if we hold the variable {Z} constant.

The confounding equivalent property was introduced previously in (Pearl, 2011). Here, we are introducing it as a test to assess if the underlying assumptions are satisfied. Assume a causal network of five variables {E,F,Z,X,Y} is identified, e.g., Figure 4A. From the network, sets {E, Z} and {F, Z } are confounding-equivalent for the identification of the causal effect of X on Y , and therefore, either of the sets is sufficient for this purpose. This means the estimated effect of X on Y based on either of these two sets do not vary significantly, which can be assessed by a statistical test. If this is not the case, the causal network is not stable and is an indicator of violation of underlying assumptions.

**Variable-Reduction Test.** In an identified causal network, consider a node that has only a role as a response/receptor. The property of this node is no arrows out but arrows in, such as node Y in Figure 4A. From the network, we conclude that the corresponding variable to node Y does not affect any other variables. Therefore, if we remove the corresponding variable from the set of variables, and then, identify a causal network of the subset, we expect the relationships among the variables in the subset to stay the same as before, see the decomposition of the joint probability distribution in Supplementary.

To quantify this in practice, we can use the receiver operating characteristic (ROC) curve for a different number of nodes having only a role as a response/receptor in the network (Yazdani et al., 2020). If this test leads to an unstable result, the underlying MR assumptions, while identifying the causal network, are violated.

**Variable-Increment test.** Assume we are interested in the effect of X on Y in an identified causal network, and Z is the set of confounders identified graphically. For T∉Z , we conclude either Y⊥T|(X,Z) or X⊥T|Z .

Considering Figure 4B, and the additive assumption to estimate the effect of X on Y , we expect the equality ${\beta_{\;1}=\beta^{\prime }}_{1}$ from $y={{\;\beta }_{\;0}+\beta }_{\;1}\;x{+\beta }_{\;2}z+e_{y}$ and $y=\beta^{\prime }{+\beta^{\prime }}_{1}\;x{+\beta^{\prime }}_{2}z{+\beta^{\prime }}_{3}t+e_{y}$ since the asymptotic bias produced by these two equations is the same. The equality ${\beta_{\;1}=\beta^{\prime }}_{1}$ can be assessed by a statistical test, such as a Z -test.

**Permutation Test**. Permutation analysis can be performed to examine the stability of an identified causal network. Since the implementation of the permutation test for causal networks is not straightforward, here, we review how to perform a permutation test to assess the stability of a causal network (Yazdani et al., 2020): the sets of randomly selected nodes for permutation must be entirely from the receptors (no arrows out but arrows in) or broadcasters (no arrows in but arrows out) since the impact of receptors and broadcasters in the network are different. For each permutation, we may select different numbers of nodes for permutation depending on the size of the network, e.g., 10 nodes, that all have the same number of arrows out (out-degree) and the same number of arrows in (in-degree). Then, permute the nodes and after that, assess the stability of the identified connections using the ROC curve.

### Identification of causal networks in real data

In this section, we review an application to identify a causal network in real data. We review a study of systematic integration of genetics and metabolomics to identify the metabolomic-causal network (Yazdani et al., 2016b). Metabolomic and genomic data were available for 2,479 individuals. **First**, we adjusted metabolites for the set of covariates in the study, such as age, sex, and body mass index. **Second**, we selected a set of IV s with a strong association with the metabolites in the study. These IVs can be identified through a metabolite QTL study, and/or by generating polygenic factors. We did not remove from the study the entities with no strong IV s. In the **third** step, we assessed conditional independence properties among metabolites using a constraint-based Bayesian algorithm. Note that this step can be carried out using a score-based algorithm too. **Fourth**, for each of the conditional dependence properties from step 3, we assessed the exclusive effect of the IV of a metabolite (as an explanatory variable) on the other metabolite (as a response). If the effect is not significant, the lack of pleiotropy assumption is satisfied. After selecting valid IV s through steps 2 and 4, we assess the causal effect of a metabolite on the other metabolite. These steps were embedded in the G-DAG algorithm (Yazdani et al., 2016a), and the metabolomic-causal network of 122 metabolites was identified using 325 valid IV where the tuning parameter was set equal to 0.001 determined by minimizing the average Hamming Distance (Tsamardinos et al., 2006), Figure 5A.

**FIGURE 5:**
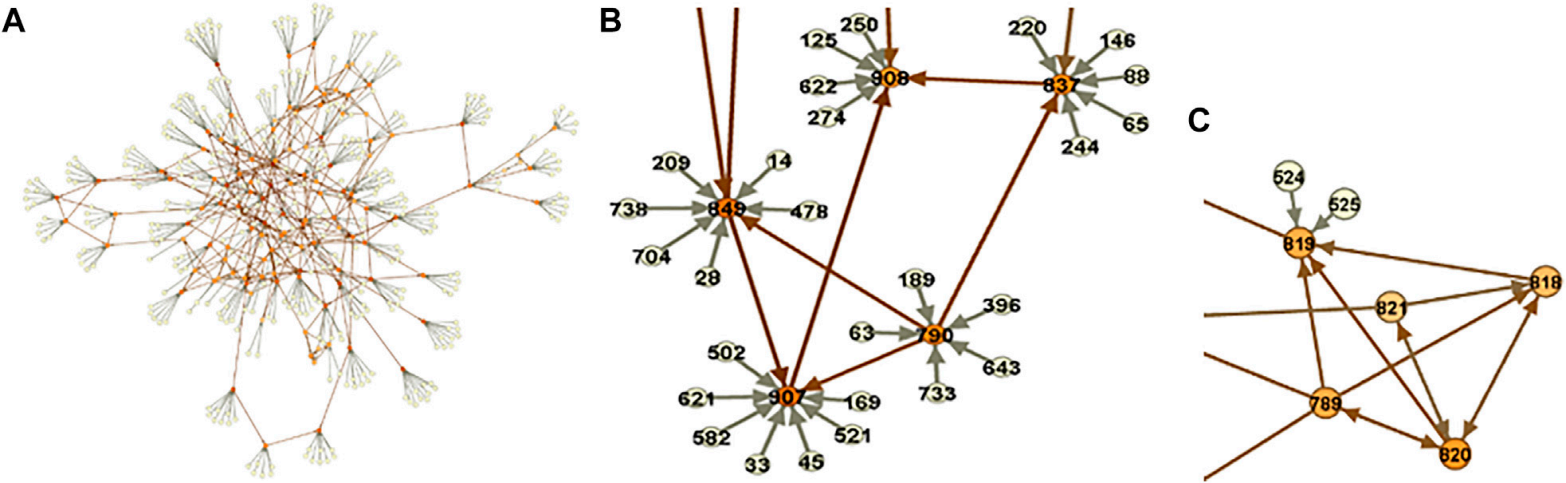
Metabolomic-causal network. **(A)**. In total, 325 polygenic factors satisfied MR assumptions/valid IVs (pale nodes) and were used to facilitate the identification of the causal network of 122 metabolites (orange nodes). **(B).** A close-up of the network. **(C).** A part of the network with no genome IV a result, some of the causal relationships are not identified, depicted as bi-directed links. Interestingly, we noticed that the corresponding metabolites are dietary-related metabolites that are mostly influenced by environmental factors and not genetics.

A close-up of the network is depicted in Figure 5B. We see that using IV s (pale nodes) facilitated the identification of causal relationships among metabolites (orange nodes). However, there was a part of the network, where no IV was identified for the metabolites and therefore, no causal conclusion could be made, depicted as bi-directed links in Figure 5C. Interestingly, these metabolites are diet-related metabolites and therefore, influenced mostly by environmental factors and not genetics and that is the reason that no genetic IV s were identified for them.

**FIGURE 6:**
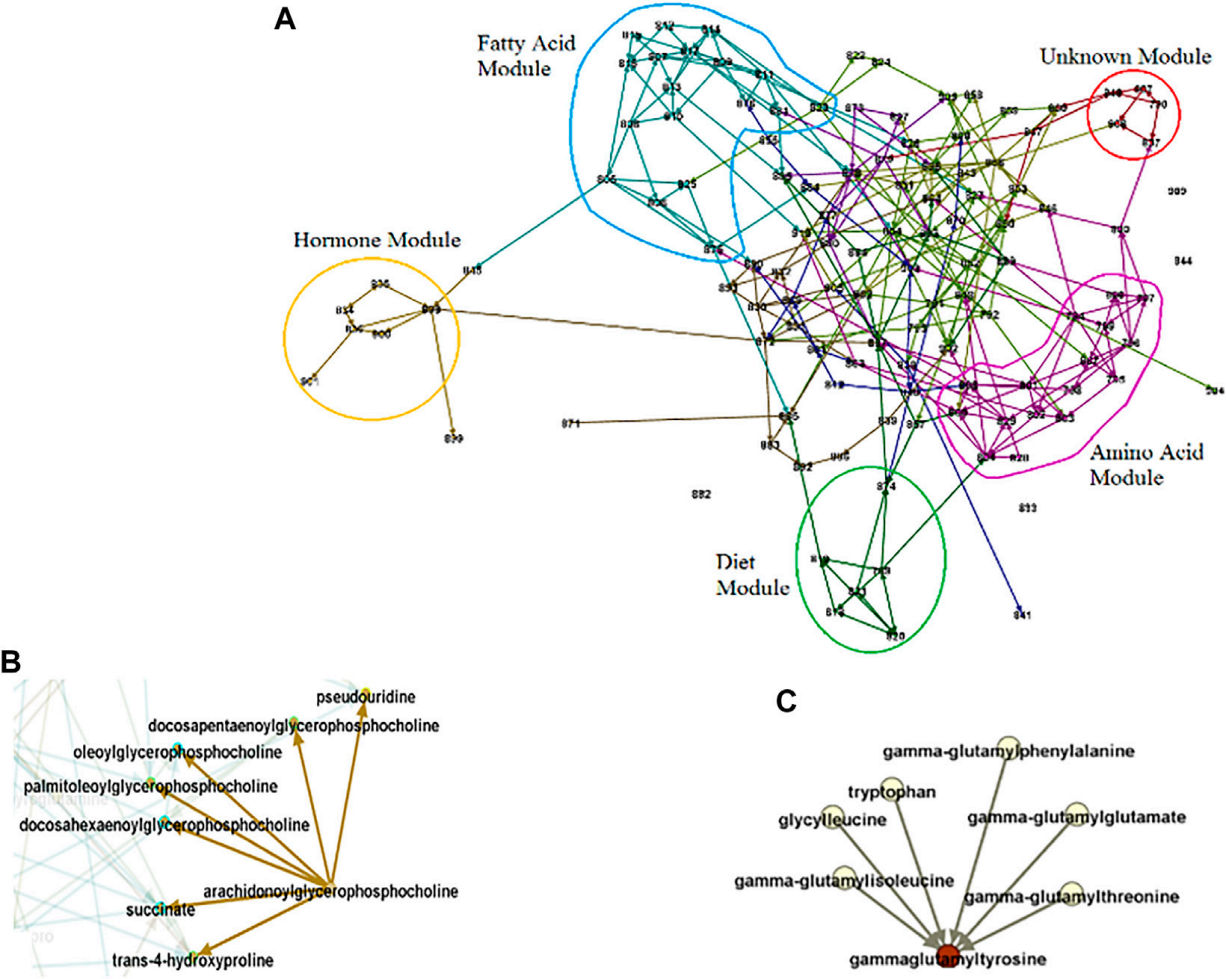
Causal Network Parameters. Numbers stand for metabolites, edges for conditional dependence properties, and arrows for causal relationships. **(A).** Modules. The set of entities that highly interact. The identified modules generally coincide with known pathways. For example, the blue and pink circles consist of related fatty acid and amino acid molecules respectively. **(B).** Example of a broadcaster. Intervention in broadcasters may change the level in the entire system since they directly or indirectly influence multiple other entities in system. **(C).** Example of a receptor. The level of receptors may predict the level of the entire system since they capture the effect of multiple other entities.

We may not be able to identify causal relationships between metabolites with no, however, having them in the analysis provides us with an opportunity to reveal the relationship with other modules or metabolites in the network (Figure 6A). Extracting information from the metabolomic-causal network and further applications are briefly reviewed in the next section as utilities of causal networks.

### The utilities of causal networks

The utilities of causal networks are multiple and flourishing, such as revealing principles governing omics under study and understanding them as a system, understanding functional links, explaining the results of perturbations, as well as facilitating efficient experimental/clinical designs. We here review the utilities by exploring the metabolomic-causal network identified in the previous section as well as using the network for downstream analysis.

In addition to effect size and significance level for each entity, using causal networks, we can reveal the role of entities in the system under study. Through exploring the network, we can identify modules/sub-networks, a set of entities that interact with each other to control a specific function (Figure 6A). The border of each module is determined using causal effect size and the in-degree and out-degree of nodes (Yazdani et al., 2016b). Each of the modules in a network can be explored to understand the module as a sub-network and reveal the metabolites with essential roles. For example, for exploring the fatty acid module in Figure 6, and the dietary hypotheses made by exploring the module see (Yazdani et al., 2016c). In addition to the property of entities as a group, we can also extract information about individual entities. For example, in a causal network, we are able to identify if a hub (a highly connected node) is an entity that significantly influences the system (a broadcaster) or is significantly influenced by the system (a receptor), or is a combination of both (Yazdani et al., 2016b; Yazdani et al., 2019; Yazdani et al., 2020). Broadcasters can be seen as targets for intervention to change the level of entities in the system. On the other hand, receptors can be seen as targets to predict the level of the whole system under study (Figures 6B,C). Note that the identification of the role of a hub in the system and distinguishing between receptors and broadcasters are possible only through causal networks. Other causal network parameters can be measured to better understand the system under study, such as the effect blocking steps and the strength. Interested readers are referred to (Yazdani et al., 2016b).

**FIGURE 7:**
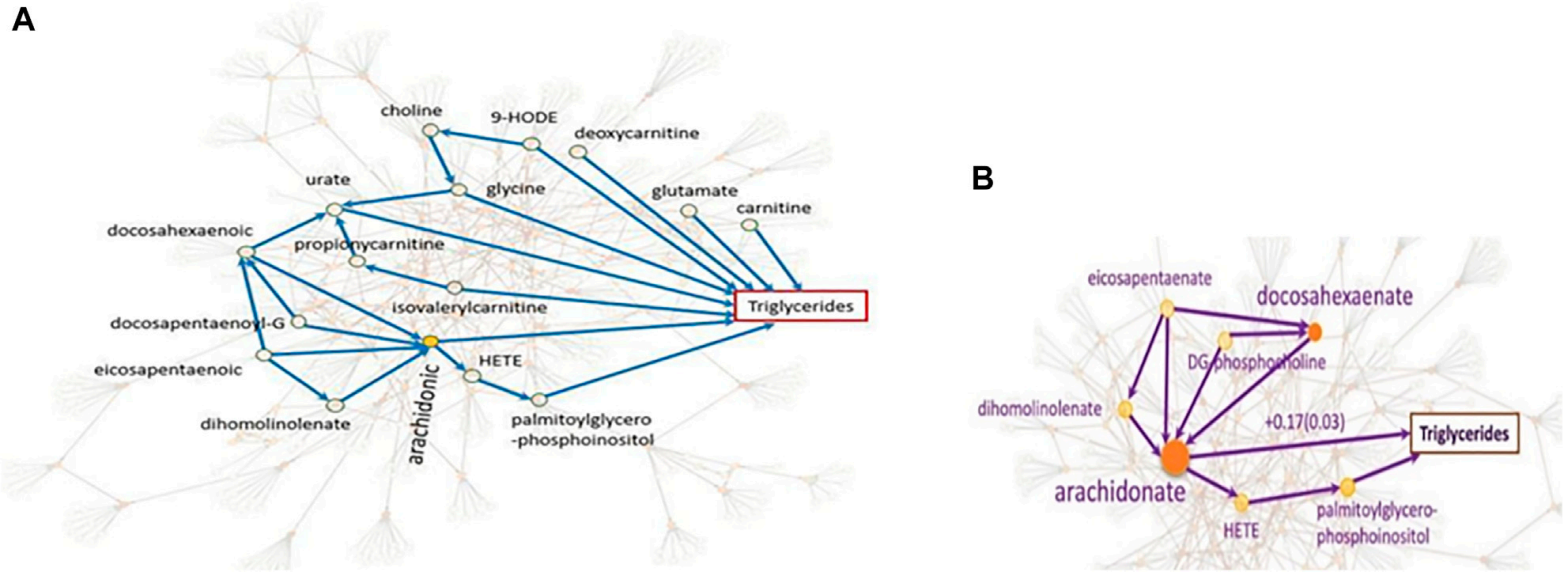
Systematic integration of genetics, metabolomics, and triglycerides. **(A).** The focus is on the nine metabolites with direct effects on triglycerides as well as some of the indirect effects. For example, no need to know about the levels of choline if we know about glycine levels since the effect of choline on triglycerides is only through glycine. **(B).** We see that the effect of four metabolites on triglycerides is through arachidonate with the largest effect on triglycerides (Yazdani et al., 2016d).

The causal networks do not only lead to a deeper understanding of how the metabolites affect each other, but also serve as the basis for downstream analyses. We explain this utility with the systematic integration of the metabolomic-causal network with triglycerides, a known risk factor for cardiovascular disease (Yazdani et al., 2016d): In total, nine out of 122 metabolites in the study were identified with a direct effect on triglyceride levels (Figure 7A). Some of the novel findings of this study were against common beliefs, such as the positive and the largest effect of arachidonate on triglycerides, which was later validated clinically (Yazdani et al., 2018) (Figure 7B). Revealing the underlying relationships facilitated efficient experimental/clinical designs. For example, in Figure 7B, we see that four metabolites exert an effect on arachidonate, and the latter has the largest effect on triglycerides. Another example is the effect of choline on triglycerides that is through glycine therefore, no need to know about the levels of choline if we know about glycine levels.

The other utility of causal networks is improving biological understanding of the GWAS pathways leading to disease (Ainsworth et al., 2017). The gene/protein *KIAA1755* with an unknown function is identified with a strong relationship with metabolite eicosapentaenoate which affects essential hypertension with no known cause (Yazdani et al., 2019). Mapping the GWAS finding on the metabolomic-causal network revealed that this metabolite was among four metabolites with a high impact on arachidonic acid with the greatest positive impact on triglyceride levels. This finding has been clinically validated (Yazdani et al., 2018). The relationship between triglycerides and essential hypertension has also been validated in a clinical study (Turak et al., 2016; Catanzaro et al., 2021). These findings may reveal new avenues into gene functional annotation and the understanding of the disease etiology.

The last utility of causal networks that we will review here is assessing GWAS findings hypothesized with pleiotropy. Causal networks reveal the underlying relationships, therefore, providing an opportunity to satisfy the assumptions of structural equation modeling and assess if a GWAS finding affects two entities independently or if it is just an indirect effect (Yazdani et al., 2019).

## Discussions

A key challenge for elucidating disease mechanisms in the 21st Century is understanding the topology and dynamics of molecules (Kim et al., 2010). Systematic integration of multi-omic data enables us to illuminate the underlying molecular networks. Despite this potential, the dominant approach is studying individual components one at a time. Complex mechanisms that use multiple omics cannot be understood by finding one causal factor. While finding one causal relationship is one step further in association studies and we achieve some understanding in this way, progress is limited because it does not provide a complete context to interpret the findings (Zhu et al., 2012). Developing systems approaches are required to bridge data analysis to the mechanistic understanding of diseases.

Identification of causal networks, as a systematic analysis of data, is established in the recognition of the hierarchical structure of the biological systems and reflects the underlying patterns (Barabási and Oltvai, 2004). The application of causal networks provides a path to uncover the role of each entity in a system, as well as providing global insights that give us a deep understanding for discovery. By mining causal networks, we can identify the role of each entity and distinguish intervention targets from prediction (Pearl, 2011). In addition, using causal networks, we can uncover groups of entities that work together to perform a certain function.

MR techniques can be modulated for systematic analysis of large-scale omics and identification of molecular networks. Some recent discussions toward this goal can be found in (Ainsworth et al., 2017; Howey et al., 2020). Opposite to classical MR approaches that are hypothesis-based, causal networks are discovery-based which makes them suitable for omic data integration where we face a large set of entities and have little knowledge about underlying relationships. Extracting information from genetic variants to generate polygenic factors and utilize them as IVs facilitates the identification of causal networks on large scale. Although there is an increasing number of applications of causal networks, more innovative approaches are required to modulate MR for integrating omics systematically, such as the identification of causal networks based on summary statistics. In addition, the research on the identification of causal networks can be extended to the integration of multiple intermediate omics.
