# Supplementary material for: From classical mendelian randomization to causal networks for systematic integration of multi-omics
Source: Front Genet. 2022 Sep 15;13:990486. doi: 10.3389/fgene.2022.990486 (PMC9520987; doi:10.3389/fgene.2022.990486)
Supplement: Supplementary file 1 [file Table1.DOCX]

**Supplementary**

**Construction of Causal networks**

Content

1. Causal networks and a formal definition

2. Markov equivalent properties

3. Application of exogenous variables such as instrumental variables

4. Conflicts in v-structure identification

5. Number of observations/Sample size

6. Back-door criterion and the tests to assess the stability and accuracy of an estimated causal network

**1. Causal networks and a formal definition.** In practice and real data applications, the concept of causal inference is best visualized as Bayesian Networks augmented with instrumental variables ($IV$s). A Bayesian network is a probabilistic graphical model that represents a set of variables and their conditional dependencies via a directed acyclic graph (DAG). But such a $DAG$ is not stable unless we use external information such as instrumental variables ($IV$s). A Bayesian network represents a multivariate distribution in a convenient and accessible form as a graph. But a causal network (i.e., $DAG$) is beyond that; it represents not only the distribution of the observed variables but also distributions under external interventions. Compatible with $DAG$s is structural equation modeling (SEM), the most applied method and pragmatic for the large-scale analysis. The popularity of these methods derives, in part, from the structure of these models that is well suited to algorithm development and in another part because the network representation of these models enables predictions under hypothetical interventions and construction of specific and strong hypotheses, which is important for decision making in many fields including biology specifically when we are facing many variables.

Assume $DAG D=(v,\varepsilon)$; $v$ is a set of random variables represented by nodes in $DAG D$ and $\varepsilon$ is a set of edges between the variables. Assume $P$ is a joint probability distribution on $v$. $D$ and $P$ must satisfy the Markov condition [1]: every variable, $Y_{i}\in v$, is independent of any subset of its predecessors conditioned on the set of direct causes or parents of $Y_{i}$, *pa*(*i*):

$Y_{i}\perp\left\{ Y_{k};i\&k\in v\backslash pa\left( i \right) \right\}|Y_{pa(i)}$.

whereas $Y_{k}$ occurs before $Y_{i} ,$ and parental set denotes the set of immediate causes (parents) of node $i$ relative to the underlying structure/ true $DAG D$*.* Joint probability over set $v$ of $DAG D=(v,\varepsilon)$ is factorized as following:

$P(Y_{1},...,Y_{p})=\prod_{i=1}^{p} P(Y_{i}|Y_{pa(i)})$, (1)

whereas $i=1, \ldots, p$ stands for the variables in set $v$ and $Y_{pa(i)}$ stands for the set of immediate causes or parents of $Y_{i}$ identified from $DAG D=(v,\varepsilon)$. Considering a deterministic/functional relationship among variables, we have the following SEM

$Y_{i}=f_{i}(Y_{pa(i)},U_{i})$, for $i=1,...,p$, (2)

where $U_{i}$ stands for all the factors that affect $Y_{i}$ when $Y_{pa(i)}$ is held constant. The only variable that is considered random is $U_{i}$ (i.e. the entire randomness in this non-deterministic model is confined in $U_{i}$), which is an extra underlying assumption in functional models [2].

Under Gaussian assumption, SEM is linear

$Y_{i}=BY_{pa(i)}+U_{i}$, for *i* = 1, …, *p*. (3)

where $B$ is a $1\times q$ vector including coefficients of $Y_{pa(i)}$ which is a $q\times1$ vector.

In a causal network, the existence of a directed edge *Y* $\bullet\to\bullet$*Z*, or simply $Y\to Z$, means that $Y$ may have a direct effect on $Z$, and the absence of a directed edge from $Z$ to $Y$ means that $Z$ does not have a direct effect on $Y$. The concept of a causal network $D=(v,\varepsilon)$ depends on the variables $v$ and edges $\varepsilon$, and, therefore, any inference depends on the set $D=(v,\varepsilon)$. A single-headed arrow indicates the possibility of a causal connection, the strength of which remains to be estimated from the data.

Identifying $DAG$s from a data set begins with identifying dependencies among the variables, which is illustrated graphically by links or lack of links between variables.

**2. Markov equivalent property.** Conditional dependencies are symmetric: when $X\perp Y$, we conclude $Y\perp X$. However, symmetry is not a property of causal relationships: we cannot conclude that $Y$ is a cause of $X$ because $X$ is a cause of $Y$. As a simple illustration, consider three variables,, and . Assume from data, we obtain $Y_{1}\perp Y_{2}$, as well as
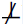
; graphically *
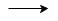
*
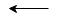
which is a unique identification of the causal relationship and is called $V$-structure, two converging arrows whose tails are not connected. Now assume, we obtain . This information provides only the skeleton among these three variables and is represented graphically as
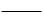

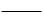
*,* which can be illustrated by three different causal networks which all are Markov equivalent DAGs:


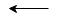

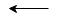
 *,*
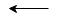
*
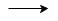
* *,* or*
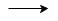
**
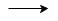
**.*

A Markov equivalent class of $DAG$s comprises more than one $DAG$ so that all represent the same probability distribution but different causal relationships in some parts of the structure, and one of them is the underlying $DAG$. If the number of variables grows, the number of Markov equivalent $DAG$s ($ME DAG$s) grows considerably [3]. Therefore, after finding conditional dependencies, identification of direction is required to have a causal network. This latter step distinguishes causal inference from the association.

There are machine learning algorithms for the identification of causal networks, which are stratified into two categories, constraint-based and score-based algorithms [4], there are also hybrid methods that are a combination of these two algorithms [5]. Regardless of the novelty of the algorithms, it is not possible to identify robust directions solely based on data due to the Markov property or statistically indistinguishable models. For conditions where directions cannot be identified see [6, 7]. These problems in identifying directions exist in theory and as a result, in statistics, where investigators must incorporate only a sample of the population.

If an algorithm performs well, we can only claim:

, (4)

where is a set including $ME DAG$s estimated over *n* sample and $DAG_{T}$ stands for the true/ underlying $DAG$ [8]. The number of required samples depends on the number of nodes/variables and the effect size (z-score) as well as the sparsity/density of the underlying $DAG$. We will discuss these points later.

**3. Application of exogenous variables such as instrumental variables.** Claiming that a relationship is causal requires support beyond the data in hand. Otherwise, it would not be less problematic in SEM than it is in a single equation model [9]. Exogenous variables (e.g., instrumental variables) and interventions are helpful to identify directionality [9-11]. Exogenous variables influence variables in the set $v$ while their effects are well understood. Including this information helps to identify directions among variables in the set $v$ through data analysis and reduce the set of $ME DAG$s. Assume $Y_{1}$ is an exogenous variable. Knowing $Y_{1}\to Y_{2}$ helps identify the direction between $Y_{2}$ and $Y_{3}$ from data. If $Y_{3}\perp Y_{1}|Y_{2}$, we conclude that $Y_{2}$ causes $Y_{3}$, which graphically is depicted as $Y_{2}\to Y_{3}$. If $Y_{3}$ and $Y_{1}$are independent, but they are dependent given $Y_{2}$, then $Y_{3}$ causes $Y_{2}$, which graphically is depicted as $Y_{3}\to Y_{2}$.

A simulation is reviewed below to highlight the benefit of exogenous variables. Consider the underlying $DAG$ in Figure 1 and simulate some data over these 5 variables. The estimated $DAG$ based on the simulated data (Figure 1) is a class of $ME DAG$s including the underlying $DAG$.


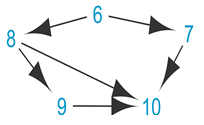

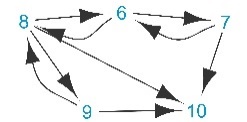


True/Underlying $DAG$ Estimated $DAG$

**Figure 1.** **Estimating a true $\boldsymbol{DAG}$ using data.** Some data is simulated based on the true $DAG$. Then, using the data, the $DAG$ on the right side is estimated. We see that multiple $ME DAG$s are estimated while estimating an underlying $DAG$ using data and without including any knowledge.

Now, we consider two exogenous variables for nodes 7 and 8, simulate some data, and then, estimate the $DAG$*,* Figure 2. We see that the estimated $DAG$ is completely oriented which is due to considering the two exogenous variables 2 and 3 for nodes 7 and 8.


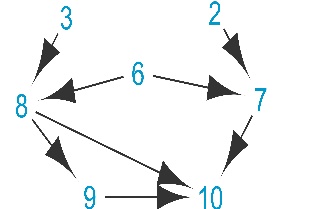

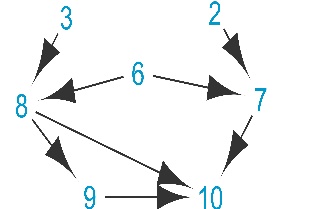


True/Underlying $DAG$ Estimated $DAG$

**Figure 2.** **Estimating a true $\boldsymbol{DAG}$ using data** **and exogenous variables**. The two variables 2 and 3 are exogenous variables. Having this knowledge and using the data simulated based on the true $DAG$, the true $DAG$ is estimated perfectly. This is the advantage of exogenous variables which facilitate the identification of directions.

If the exogenous variables are genetic information (e.g., genetic variants), this identification is called Mendelian randomization (MR) since it is based on the principles of MR.

**4. Conflicts in $\boldsymbol{V}$-structure identification.** $V$-structures make the probability distributions distinguishable, therefore, they are identifiable from data as mentioned earlier. However, finding $V$-structures may end in a conflict, which is estimating two different directions between two variables. For example, in Figure 3.a, there is a conflict in direction estimation between $Z$ and $D$ due to the estimated $V$-structures $G\to D\leftarrow Z$ and $H\to Z\leftarrow D$*.* In the existence of this conflict, some algorithms select one of the directions randomly instead of determining the area of conflict. However, this will be problematic. Here, we delve into the problem.

a
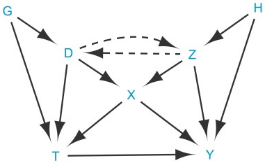
 b
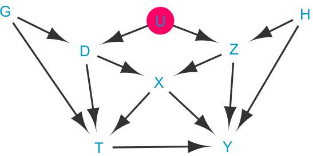
 c
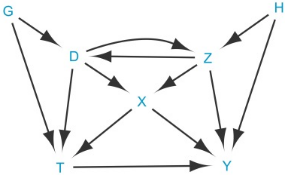


d
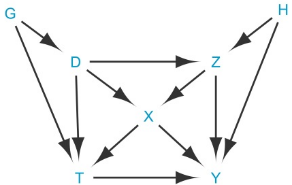

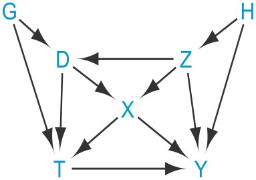


Random Graph 1 Random Graph 2

**Figure 3. Conflict in $\boldsymbol{V}$-structure identification.** **a.** While estimating a network, a conflict occurs in the estimation of the direction between $D$ and $Z$. **b.** The case that the conflict is due to an unobserved confounder $U$. **c.** The case that the conflict is due to a loop. **d.** Two possible random $DAG$s of “a”, which is selecting one of the possibilities in “a” without delving into the conflict. If we select either of the random $DAG$s, the effect $T$ on $Y$ is confounded.

Assume our interest is finding the effect $T$ on $Y$, and there is a conflict in finding the relationship between $D$ and $Z$, Figure 3. a. One scenario is the conflict is due to an unobserved variable $U$ affecting both $D$ and $Z$, Figure 3. b. Therefore, $Z$ and $D$ are dependent through $U$, and there are two $V$-structures, one in $D$ and the other in $Z$. In this case, to find the effect $T$ on $Y$, either of the two sets of confounders $S_{1}=\{X,Z,H\}$ or $S_{2}=\{X,D,G\}$. is sufficient. This means by fixing $S$(either $S_{1}$ or $S_{2}$), the effect of any changes in $T$ to $Y$ is directly through $T$. In the present of a cycle, Figure 3. c, the sufficient set of confounders are the same.

If instead of delving into the conflict as above, we choose one of the random $DAG$s, Figure 3.d, the study of effect $T$ on $Y$ is confounded. In random $DAG$ 1, the set $S=\{X,D\}$ of confounders seems sufficient. However, conditioning on the set $S$ opens the indirect path $T\leftarrow G\to D\leftarrow U\to Z\to Y$ between $T$ and $Y$. In random $DAG$ 2, the set $S=\{X,Z\}$ of confounders seems sufficient. However, conditioning on this set opens the indirect path $T\leftarrow D\leftarrow U\to Z\leftarrow H\to Y$ between $T$ and $Y$. Therefore, in both cases of random $DAG$s, the study is confounded because the $IV$ assumption is violated.

**5. Number of observations/Sample size.** In applications, the stability of the estimated causal networks depends on the sample size in addition to other factors reviewed so far. Here, we review this point through a simulation study. Consider the underlying $DAG$ in Figure 4. The joint probability distribution over these 10 variables is distinguishable except between variables 3 and 1, which are two $ME DAG$s. Therefore, the causal relationship between 1 and 3 cannot be determined from data regardless of the sample size. However, the role of sample size is in the identification of the $ME$ class that includes the underlying $DAG$.

To estimate the $ME$ class accurately, 900 samples are sufficient. But, with fewer samples, e.g., 700 samples, the $V$-structure at 5 cannot be detected. Using 500 samples, a new $V$-structure appears at 8, $6\to8\leftarrow9$. Both the undetected $V$-structure (Figure 4., $n$ = 700) and the false $V$-structure (Figure 4., $n$ = 500 samples) create new Markov equivalent classes which do not contain the underlying $DAG$.


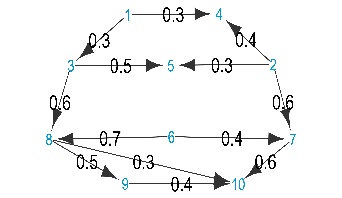

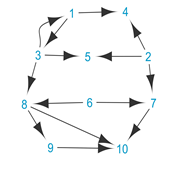

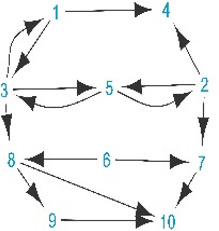

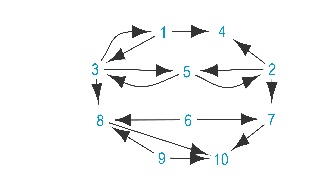


True/Underlying $DAG$ $n$ = 900 $n$ = 700 $n$ = 500

**Figure 4**. **Sample size and estimating a $\boldsymbol{DAG}$.** Estimating an underlying $DAG$ using different sample sizes. With 900 samples, the $ME DAG$ of the true $DAG$ is estimated. With 700 samples, a $V$-structure in node 5 is not identified. With 500 samples, a wrong $V$-structure at node 8 is estimated.

The required sample size depends on the strengths of the effects as well as the number of variables and sparsity of the underlying $DAG$. In figure 4, the effect sizes vary in the range $[0.3, 0.7]$ and 900 samples were required to identify the $ME$ class accurately. In figure 5, we increase the effect sizes to the range $[0.8, 2.6]$ and then simulate some data. In this case, even 500 samples are sufficient to estimate the $ME$ class accurately.


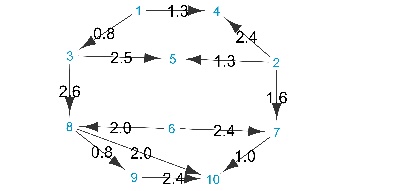

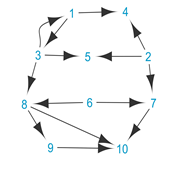


True/Underlying $DAG$ n=500

**Figure 5**. **Effect size and estimating a $\boldsymbol{DAG}$.** The larger effect size depicted in the left network, compared to Figure 4., requires less sample to estimate the $ME DAG$s of underlying $DAG$. With 500 samples the $ME DAG$ of the true $DAG$ is identified because the effect sizes are larger.

The required samples to estimate $ME DAG$s depend on the number of variables in the study, effect sizes, and the sparsity of the underlying network, which are mostly unknown. Therefore, to determine the accuracy of an estimated network, we apply some assessments explained below.

**6. Back-door criterion and the tests to assess the stability and accuracy of an estimated causal network.**

Assume we are given a causal network $D$. On a subset $v$ of the observed variables in $D$, we wish to estimate the causal effect of $X$ on a set of response variables $Y$, where $X$ and $Y$ are two subsets of $v$. Using the back-door criterion, we can identify the sufficient set of variables (i.e., confounders) for identification of the causal effect of $X$ on $Y$ [1, 12].

**Back-door criterion.** A set of variables $Z$ satisfies the cack-door criterion relative to an ordered pair of variables $(X_{i}, X_{j})$, in a $DAG D$ if:

1. No node in $Z$ is a descendant of $X_{i}$, and
2. $Z$ blocks every path between $X_{i}$ and $X_{j}$ that contains an arrow into $X_{i}$.

**Variable Reduction Test.** Assume is estimated over the set $v$ and there is a so that for . Define $v^{*}$ and estimate causal structure over the set $v^{*}$. If the estimated causal structure over the reduced set, , is not the same as, the structure is not robust. If is the same as , the test is carried out again on .

stands for the estimation of the causal structure of set $v$ by application of $n$ sample, stands for estimated causal structure over a reduced set $v^{*}$, $v\backslash j$ means node $j$ is eliminated from the estimated structure, and means the edges which coming to node $j$ are eliminated.

Assume in the set of variables of the causal network $D$, variable $Y_{p}$ does not influence other variables based on $P$*, i.e.,* $Y_{p}$ has a role only as a response variable. Therefore, the joint probability distribution $P$ is factorized as

$P(Y_{1},...,Y_{p})=P(Y_{p}|Y_{pa(p)})P(Y_{1},...,Y_{p-1})$. (6)

As seen, the probability distribution of variables $Y_{1},...,Y_{p-1}$ is independent of variable $Y_{p}$. Therefore, removing $Y_{p}$ from the set of variables is not expected to affect the estimation of the causal network of the remaining set of variables.

**Variable-Increment test.** This test is also based on decomposition of joint probability distribution of the set of variables in a causal network. In the decomposition of probability distribution in (6), consider the factor $P(Y_{p}|Y_{pa(p)})$. All variables with a direct effect on $Y_{p}$ (i.e., $Y_{pa(p)}$) are in the condition. In the condition, adding a set of predecessors of $Y_{p}$ in addition to $Y_{pa(p)}$ will produce the same asymptotic bias relative to the target quantity.

**References:**

1. Pearl, J., *Causality: Models, reasoning, and inference, second edition*, in *Causality: Models, Reasoning, and Inference, Second Edition*. 2011.

2. Dawid, A.P., *Fundamentals of statistical causality*, in *RSS/EPSRC Graduate Training Program*. 2007. p. 1-94.

3. Kalisch, M. and P. Bühlman, *Estimating high-dimensional directed acyclic graphs with the PC-algorithm.* Journal of Machine Learning Research, 2007. **8**(3).

4. Acid, S., et al., *A comparison of learning algorithms for Bayesian networks: a case study based on data from an emergency medical service.* Artificial intelligence in medicine, 2004. **30**(3): p. 215-232.

5. Murphy, K.P., *Inference and learning in hybrid Bayesian networks*. 1998: Citeseer.

6. Isozaki, T. and M. Kuroki, *Learning Causal Graphs with Latent Confounders in Weak Faithfulness Violations.* New Generation Computing, 2017. **35**(1): p. 29-45.

7. Verma, T. and J. Pearl. *An algorithm for deciding if a set of observed independencies has a causal explanation*. in *Uncertainty in artificial intelligence*. 1992. Elsevier.

8. Hauser, A. and P. Bühlmann, *Characterization and greedy learning of interventional Markov equivalence classes of directed acyclic graphs.* The Journal of Machine Learning Research, 2012. **13**(1): p. 2409-2464.

9. Dawid, A.P. *Beware of the DAG!* in *Causality: objectives and assessment*. 2010. PMLR.

10. Pearl, J., *Causality*. 2009: Cambridge university press.

11. Heckman, J. and S. Navarro-Lozano, *Using matching, instrumental variables, and control functions to estimate economic choice models.* Review of Economics and statistics, 2004. **86**(1): p. 30-57.

12. Yazdani, A., A. Yazdani, and E. Boerwinkle, *Conceptual aspects of causal networks in an applied context.* J Data Mining Genomics Proteomics, 2016. **7**(02): p. 2-4.
